# Galectin-3 Silencing Inhibits Epirubicin-Induced ATP Binding Cassette Transporters and Activates the Mitochondrial Apoptosis Pathway via β-Catenin/GSK-3β Modulation in Colorectal Carcinoma

**DOI:** 10.1371/journal.pone.0082478

**Published:** 2013-11-26

**Authors:** Yung-Kuo Lee, Tsung-Hsien Lin, Chuan-Fa Chang, Yu-Li Lo

**Affiliations:** 1 Institute of Basic Medical Science, College of Medicine, National Cheng Kung University, Tainan, Taiwan; 2 Department of Medical Laboratory Science and Biotechnology, College of Medicine, National Cheng Kung University, Tainan, Taiwan; 3 Center of Infectious Disease and Signaling Research, College of Medicine, National Cheng Kung University, Tainan, Taiwan; 4 Department of Biological Sciences and Technology, National University of Tainan, Tainan, Taiwan; 5 School of Pharmacy, National Taiwan University, Taipei, Taiwan; University of Technology Sydney, Australia

## Abstract

Multidrug resistance (MDR), an unfavorable factor compromising the treatment efficacy of anticancer drugs, involves the upregulation of ATP binding cassette (ABC) transporters and induction of galectin-3 signaling. Galectin-3 plays an anti-apoptotic role in many cancer cells and regulates various pathways to activate MDR. Thus, the inhibition of galectin-3 has the potential to enhance the efficacy of the anticancer drug epirubicin. In this study, we examined the effects and mechanisms of silencing galectin-3 via RNA interference (RNAi) on the β-catenin/GSK-3β pathway in human colon adenocarcinoma Caco-2 cells. Galectin-3 knockdown increased the intracellular accumulation of epirubicin in Caco-2 cells; suppressed the mRNA expression of galectin-3, β-catenin, cyclin D1, c-myc, P-glycoprotein (P-gp), MDR-associated protein (MRP) 1, and MRP2; and downregulated the protein expression of P-gp, cyclin D1, galectin-3, β-catenin, c-Myc, and Bcl-2. Moreover, galectin-3 RNAi treatment significantly increased the mRNA level of GSK-3β, Bax, caspase-3, and caspase-9; remarkably increased the Bax-to-Bcl-2 ratio; and upregulated the GSK-3β and Bax protein expressions. Apoptosis was induced by galectin-3 RNAi and/or epirubicin as demonstrated by chromatin condensation, a higher sub-G1 phase proportion, and increased caspase-3 and caspase-9 activity, indicating an intrinsic/mitochondrial apoptosis pathway. Epirubicin-mediated resistance was effectively inhibited via galectin-3 RNAi treatment. However, these phenomena could be rescued after galectin-3 overexpression. We show for the first time that the silencing of galectin-3 sensitizes MDR cells to epirubicin by inhibiting ABC transporters and activating the mitochondrial pathway of apoptosis through modulation of the β-catenin/GSK-3β pathway in human colon cancer cells.

## Introduction

Among the various types of cancers, colorectal cancer is a serious and common health problem worldwide. This type of cancer is the third most common visceral malignancy and remains a major cause for cancer deaths due to therapy resistance [1]. The development of colorectal cancer usually occurs through a multistage process involving the mutational activation of oncogenes and inactivation of tumor suppressor genes. More than 90% of tumorigenesis in colorectal cancers begins with mutations in the β-catenin signaling pathway [2].

 Multidrug resistance (MDR), an unfavorable factor compromising the treatment efficacy of anticancer drugs, involves the upregulation of ATP binding cassette (ABC) transporters and induction of galectin-3 signaling. To circumvent MDR in cancer cells, several strategies, including P-glycoprotein (P-gp) antagonists, antisense oligonucleotides, ribozymes, and other techniques that modulate MDR-related genes, have been developed [3,4]. However, the long half-life of P-gp (at least 16 h) makes it difficult to achieve complete P-gp dysfunction [5]. To solve this problem, the transfection of MDR cells with siRNAs targeting upstream MDR-related genes in a sequence-specific manner may be promising.

Galectin-3, a β-galactoside-binding protein, exhibits a variety of biological and pathological functions, including effects on RNA processing, cell growth, differentiation, adhesion, apoptosis, immune response, malignant transformation, metastasis, and cancer drug resistance [6]. Galectin-3 is expressed in epithelial and inflammatory cells. However, there are contradictory findings regarding the over- or under-expression of galectin-3 in human colorectal cancer. Galectin-3 overexpression was observed in colorectal carcinoma, corresponding to a positive correlation with cancer progression and metastasis [7-9]. Zaia Povegliano et al. have found that a high percentage of galectin-3-stained cells could be observed in the most advanced colon cancer patients and patients with recurrence after surgery and chemotherapy treatment [10]. In contrast, a decrease in galectin-3 expression levels has also been found in colorectal cancer [11,12]. Furthermore, Greco and Iacovazzi et al. have reported that the overexpression of galectin-3 and its ligand 90K in serum could be a useful biomarker for colon cancer transformation [13,14]. Galectin-3 is an important anti-apoptotic effector protein that confers resistance to cancer chemotherapy. The knockdown of galectin-3 expression induced apoptosis in human colorectal cancer cells [9]. In addition, keratinocytes and peritoneal macrophages from galectin-3^¯/ ¯^ mice are sensitive to apoptotic stimuli relative to those cells from galectin-3^+/+^ mice [15]. After leukemia cells were stimulated for apoptosis by cisplatin, galectin-3 expression was upregulated and caused resistance to apoptosis in surviving cells [16]. Moreover, the silencing of galectin-3 with RNAi increased the susceptibility of leukemia cells to apoptosis [16].

Numerous studies focused on the molecular mechanisms of galectin-3 involved in cancer cell chemoresistance [17]. In particular, galectin-3 plays a critical role in the regulation of the expression of cancer-related genes, including cyclin D1 and Akt (also known as Protein Kinase B; PKB) [18]. Galectin-3 upregulates β-catenin expression, increases its nuclear accumulation, and augments Wnt/β-catenin signaling in human colon cancer cells by regulating glycogen synthase kinase-3β (GSK-3β) phosphorylation and activity via the PI3K/Akt pathway [19]. In addition, galectin-3 was identified as a binding partner of β-catenin and T-cell factor 4 (TCF4), which thus activates the Wnt signaling target genes cyclin D1 and c-myc in human breast cancer cells [20]. However, the role of galectin-3 in colon cancer drug resistance is complex and unclear.

In this study, we aimed to decipher the galectin-3 molecular mechanisms involved in colon cancer cell drug resistance. The expression of upstream survival signals (GSK-3β, β-catenin, c-myc and cyclin D1) in human colon adenocarcinoma Caco-2 cells was evaluated after epirubicin treatment with or without galectin-3 knockdown. The changes in downstream efflux transporters, such as P-gp (product of *MDR1* gene), MDR-associated protein (MRP) 1, and MRP2, and apoptosis-associated proteins, including Bcl-2, Bax, and caspases, were also analyzed under the same conditions. We aimed to test the possibility of RNAi directed against galectin-3 to suppress pump resistance, induce apoptosis, and enhance the chemosensitivity of Caco-2 cells to epirubicin. This is a pioneer study correlating the knockdown and overexpression of galectin-3 with ABC transporter modulation and apoptosis regulation in colon cancer cells with the MDR spectrum.

## Materials and Methods

### Reagents

Short hairpin RNA (shRNA) was purchased from the National RNAi Core Facility, Academia Sinica (Taipei, Taiwan, ROC). The shRNA sequences are listed in Table 1. The sequence of the scrambled shRNA shown as shLacZ does not target any known human gene. Epirubicin (pharmorubicin) was purchased from Pfizer, Inc. (New York, NY, USA). The Lipofectamine^®^ 2000 and PolyJet DNA transfection reagents were purchased from Invitrogen (Carlsbad, CA, USA) and SignaGen Laboratories (Ijamsville, MD, USA), respectively. All cell culture medium and reagents were purchased from Gibco (Grand Island, NY, USA) or Hyclone (Logan, UT, USA). Most of the other chemical reagents were purchased from either Merck (Darmstadt, Germany) or Sigma-Aldrich (St. Louis, MO, USA).

**Table 1:** The sequences of shRNA and primer sequences used for knock-down and real-time PCR.

Gene name	Sequences
shGal-3 target sequence	5’ -GCTCACTTTTGCAGTACAAT-3’
shLacZ target sequence	5’-TGTTCGCATTATCCGAACCAT-3’
Gal-3 forward primer	5’-TAATAACTGGGGAAGGGAAG-3’
Gal-3 reverse primer	5’-AGCACTGGTGAGGTCTATGT-3’
GSK-3β forward primer	5’-ACTTTGTGACTCAGGAGAACTGG-3’
GSK-3β reverse primer	5’-TCGCCACTCGAGTAGAAGAAATA-3’
β-catenin forward primer	5’-TCAGATCTTAGCTTACGGCAA-3’
β-catenin reverse primer	5’-TCAGATCTTAGCTTACGGCA-3’
Cyclin D1 forward primer	5’-CACACGGACTACAGGGGAGT-3’
Cyclin D1 reverse primer	5’-CACAGGAGCTGGTGTTCCAT-3’
C-myc forward primer	5’-TCAAGAGGCGAACACACAAC-3’
C-myc reverse primer	5’-GGCCTTTTCATTGTTTTCCA-3’
Bcl-2 forward primer	5’-TTCTTTGAGTTCGGTGGGGTC-3’
Bcl-2 reverse primer	5’-TGCATATTTGTTTGGGGCAGG-3’
Bax forward primer	5’-ACCAAGAAGCTGAGCGAGTGTC-3’
Bax reverse primer	5’-ACAAAGATGGTCACGGTCTG-3’
Caspase 3 forward primer	5’-GAATACCCTGGACAACA-3’
Caspase 3 reverse primer	5’-ACGCCATGTCATCATCAA-3’
Caspase 8 forward primer	5’-GGATGCCTTGATGCTATTCC-3’
Caspase 8 reverse primer	5’-TCCTTCAATTCTACTTTGTTCACATC-3’
Caspase 9 forward primer	5’-GCCATGGACGAAGCGGATCGGC-3’
Caspase 9 reverse primer	5’-GGCCTGGATGAAGAAGAGCTTGGG-3’
18s rRNA forward primer	5’-CAGAATCCACGCCAGTACAA-3’
18s rRNA reverse primer	5’-AATCTTCTTCAGTCGCTCCA-3’

### Cell lines

The Caco-2 cell line was obtained from the Bioresource Collection and Research Center of the Food Industry Research and Development Institute (Hsinchu, Taiwan). Caco-2 cells express multiple efflux transporters, including P-gp, MRP1, MRP2, and Bcl-2 [21]. This colon epithelial adenocarcinoma cell line has been used as a model cancer cell line with MDR phenotype in our lab [22,23]. Cells were cultured in Dulbecco's modified Eagle's medium (DMEM) supplemented with 20% fetal bovine serum (FBS; Hyclone), 0.1 mM nonessential amino acids, and 10,000 units/ml of penicillin/streptomycin (Gibco) at 37 °C in a humidified atmosphere of 5% CO_2_ and 95% air.

### Construction of galectin-3 shRNA

 Galectin-3 shRNA (shGal-3) was constructed using the pLKO.1-puro (Amp^+^) vector with an U6 promoter. shGal-3 plasmid DNA was extracted using a plasmid mini kit (Qiagen), and lentiviruses were generated by the RNAi Core of the Research Center of Clinical Medicine, National Cheng Kung University Hospital. Briefly, 293T cells were co-transfected with 5 μg packaging plasmid (pCMVΔR8.91), 0.5 μg envelope plasmid (pMD.G) and 5 μg pLKO.1 shRNA using Lipofectamine 2000 (Invitrogen) for 6 h. After 24 h, the supernatants containing viral particles were harvested and filtered through 0.45 mm filters. The filtered supernatant was mixed with polybrene (Sigma) and added to Caco-2 cells for transduction and subsequent overnight incubation. The transfected cells were incubated with DMEM containing puromycin (Amresco) for three days, and the positive clones were selected after confirmation by galectin-3 western blotting.

### Cytotoxicity assay

 Growth inhibition of Caco-2 cells was evaluated with an MTT (3-(4,5-dimethylthiazol-2-yl)-2,5-diphenyl tetrazolium bromide) assay. Cells (6×10^3^) were seeded in 96-well plates. After 48 h of treatment of the control and shGal-3 cells with increasing concentrations of epirubicin (0, 0.01, 0.1, 1, and 10 μg/ml), the cells were incubated with 0.2 mg/ml MTT and maintained in 5% CO_2_ in an incubator at 37 °C for an additional 4 h. Dimethyl sulfoxide (100 μl) was added to each well to solubilize the formazan, and the absorbance was measured at 540 nm using a microplate reader (Dynatech, Chantilly, VA, USA). The relative cell viability (%) was calculated by dividing the number of cells incubated with different treatments by the number of control cells. The median inhibitory concentration (IC_50_), defined as the drug concentration necessary to inhibit cell growth by 50%, was then obtained from the curve. Data were expressed as the means ± standard deviation (S.D.) of six experiments.

### Apoptosis detection assay

The Annexin V FITC Apoptosis Detection Kit (Roche, Cambridge, MA) was used. After a 48 h-treatment of the control and shGal-3 cells with or without epirubicin (1 μg/ml), staining was performed with Annexin V-propidium iodide (PI) labeling solution for 15 min at room temperature in the dark. Samples were measured using a flow cytometer (Cell Lab Quanta SC MPL; abbreviated as Quanta SC; Beckman Coulter, Fullerton, CA, USA). Data acquisition and analysis were performed using commercial software (Quanta SC). Early apoptotic cells with intact cell membranes expose phosphatidylserine and are bound to Annexin V-FITC, and these cells exclude PI (Annexin V positive, PI negative). Cells in necrotic or late apoptotic stages are Annexin V-FITC and PI positive.

### Observation of chromatin condensation using a fluorescence microscope

Control and shGal-3 cells were plated at 2 × 10^5^ cells/well in six-well plates and incubated for 48 h with or without epirubicin (1 μg/ml). The cells were collected and stained with acridine orange (10 mg/ml; Sigma). Samples were then observed using an inverted microscope (Eclipse TS-100; Nikon Co., Tokyo, Japan) equipped with a fluorescence image capture device (C-SHG; Nikon) controlled with the Image-Pro Plus software (Media Cybernetics, Inc., Bethesda, MD, USA). Fragmented nuclei and condensed chromatin characteristics were examined and compared with those of the control.

### Cell cycle analysis

 Control and shGal-3 cells at 10^5^ cells/well were incubated for 48 h with or without epirubicin (1 μg/ml). The cells were harvested by centrifugation and gently fixed with 70% ethanol at -20 °C overnight. After fixation, the cells were stained with propidium iodide (1 mg/ml) and incubated for 30 min at 25 °C in the dark. The cells were analyzed using a flow cytometer (Quanta SC). The presented results are from three individual experiments.

### Intracellular epirubicin accumulation

The functional involvement of MDR transporters in the efflux of epirubicin was evaluated using a flow cytometer. Control and shGal-3 cells were seeded in six-well plates at 2×10^5^ cells/well and allowed to attach overnight. After pretreatment with epirubicin for 48 h, the fluorescent epirubicin that accumulated in Caco-2 cells was measured using a flow cytometer (Quanta SC). Data acquisition and analysis were performed using commercial software (Quanta SC), and determinations were performed in quadruplicate.

### Construction and overexpression of galectin-3

The 741-bp human galectin-3 (*hGal-3*) full-length sequence was amplified by PCR with the following primers: 5´-GAGCGGCCGATGGCAGACAATTTTTCGCTC CA-3´ (forward) and 5´-GAGGGATCCTTATATCATGGTATATGAAGCAC-3´ (reverse). The gel-purified PCR product was digested using *Bam* HI and *Xho* I and then cloned into the pIRES-hrGFP-2a vector (Stratagene Corp., La Jolla, CA, USA) using T4 DNA ligase (Promega, Madison, WI, USA). All of the resulting plasmids were amplified in *E. coli* DH5α. Successful construction of the pIRES*-hGal-3* vector was confirmed using DNA electrophoresis and direct sequencing, which showed a similarity of 99.9%. Caco-2 cells were plated at 1 × 10^5^ cells/well in six-well plates and allowed to attach overnight. We mixed 2 μg/well of the pIRES*-hGal-3* gene constructs with 6 μl/well of the PolyJet DNA transfection reagent (SignaGen Laboratories, Ijamsville, MD, USA) in 200 μl DMEM. Caco-2 cells were then transfected with the mixture at 25 °C for 30 min and incubated at 37 °C for 48 h.

### Real-time PCR of P-gp, MRP1, MRP2, Bax, Bcl-2, and caspases

Control and shGal-3 cells were treated with or without epirubicin (1 μg/ml) for 48 h. Total RNA was extracted from cells using the Total RNA Extraction Miniprep System (Viogene, Taipei, Taiwan) according to the manufacturer’s instructions. The RNA yield and purity were assessed using a NanoDrop 2000 (Thermo, Wilmington, DE, USA). cDNA was prepared from total RNA using the High-capacity RNA-to-cDNA kit (Applied Biosystems; Foster City, CA, USA) following the manufacturer's protocol. The gene-specific primers (Table 1) for galectin-3, GSK-3ß, ß-catenin, Cyclin D1, c-myc, MDR1, MRP1, MRP2, Bcl-2, Bax, caspase-3, caspase-8, and caspase-9 were constructed by multiple sequence alignment. 18s rRNA was served as an internal control. Quantitative PCR was conducted using the StepOne Real-Time PCR system (Applied Biosystems) and SYBR Green PCR Master Mix (Applied Biosystems). The cycling program was performed as follows: denaturation at 95 °C for 10 min followed by 40 cycles of 95 °C for 15 s and 60 °C for 1 min. The specificity of the gene-specific PCR primers was verified by melting curve and agarose gel analyses. The results presented are from three individual experiments in which each sample was assayed in triplicate and normalized to the 18s rRNA level. Gene expression was calculated as the mRNA expression ratio compared with control cells.

### Western blotting

After shGal-3 and/or epirubicin treatment, proteins were extracted from harvested cell lysates, and the total protein concentration was determined with a spectrophotometer (U-2800A; Hitachi, Tokyo, Japan). Proteins were separated in a 10-12% polyacrylamide gel and transferred onto a polyvinylidene difluoride (PVDF) membrane. The membranes were separately incubated with specific antibodies for P-gp (Genetex), galectin-3 (Abcam), cyclin D1 (Cell Signaling Technology), c-myc (Epitomics), Gsk-3α/β (SantaCruz, gift from Prof. C. F. Lin), phospo-Gsk-3α/β (Ser21/9) (Cell Signaling Technology, gift from Prof. C. F. Lin), β-catenin (Cell Signaling Technology), Bcl-2 (Cell Signaling Technology, gift from Prof. C. F. Lin), Bax (BD Pharmingen, gift from Prof. C. F. Lin), and α-Tubulin (Sigma) overnight at 4 °C. These samples were then labeled for 1.5 h at room temperature with horseradish peroxidase (HRP)-conjugated goat anti-rabbit IgG for cyclinD1, c-myc, phospo-Gsk-3α/β, Bcl-2 and β-catenin and HRP-conjugated goat anti-mouse IgG for P-gp, Gal-3, Gsk-3α/β, Bax, and α-Tubulin. The detection of the signal was performed with an enhanced chemiluminescence detection kit (PerkinElmer Life Sciences, Waltham, MA). The gels were digitally photographed and scanned using a gel documentation system (UVIdoc; UVItec Limited, Cambridge, UK). The presented results are from three individual experiments.

### Caspase-3, caspase-8, and caspase-9 activity assay

Caspase-3, caspase-8, and caspase-9 activities were detected by the Caspase-Glo^®^ 3, Caspase-Glo^®^ 8, and Caspase-Glo^®^ 9 Assay Kits (Promega), respectively. Control and shGal-3 cells at a density of 2 × 10^5^ cells/well were harvested after treatment with or without 1 μg/ml of epirubicin for 48 h. Pellets were resuspended in DMEM, and a portion of the cell suspension (50 μl) was then mixed with 50 μl of the caspase-3, caspase-8 and caspase-9 reagents containing the corresponding luminogenic substrates Ac-DEVD-pNA, Ac-LETD-pNA, and Ac-LEHD-pNA, respectively, at room temperature for 30 min. Levels of released aminoluciferin luminescence were measured using a luminometer (Model MiniLumat LB9506; Berthold Technologies GmbH & Co. KG, Bad Wildbad, Germany).

### Statistical analysis

Data are presented as the means ± standard deviation. Student’s *t*-test was used to analyze differences between two treatment groups. Statistical analysis was conducted using one-way ANOVA and Dunnett's multiple comparison tests. Significant difference was set at P < 0.05.

## Results

### Combined epirubicin and shGal-3 treatment significantly intensified epirubicin cytotoxicity

**Figure 1:**
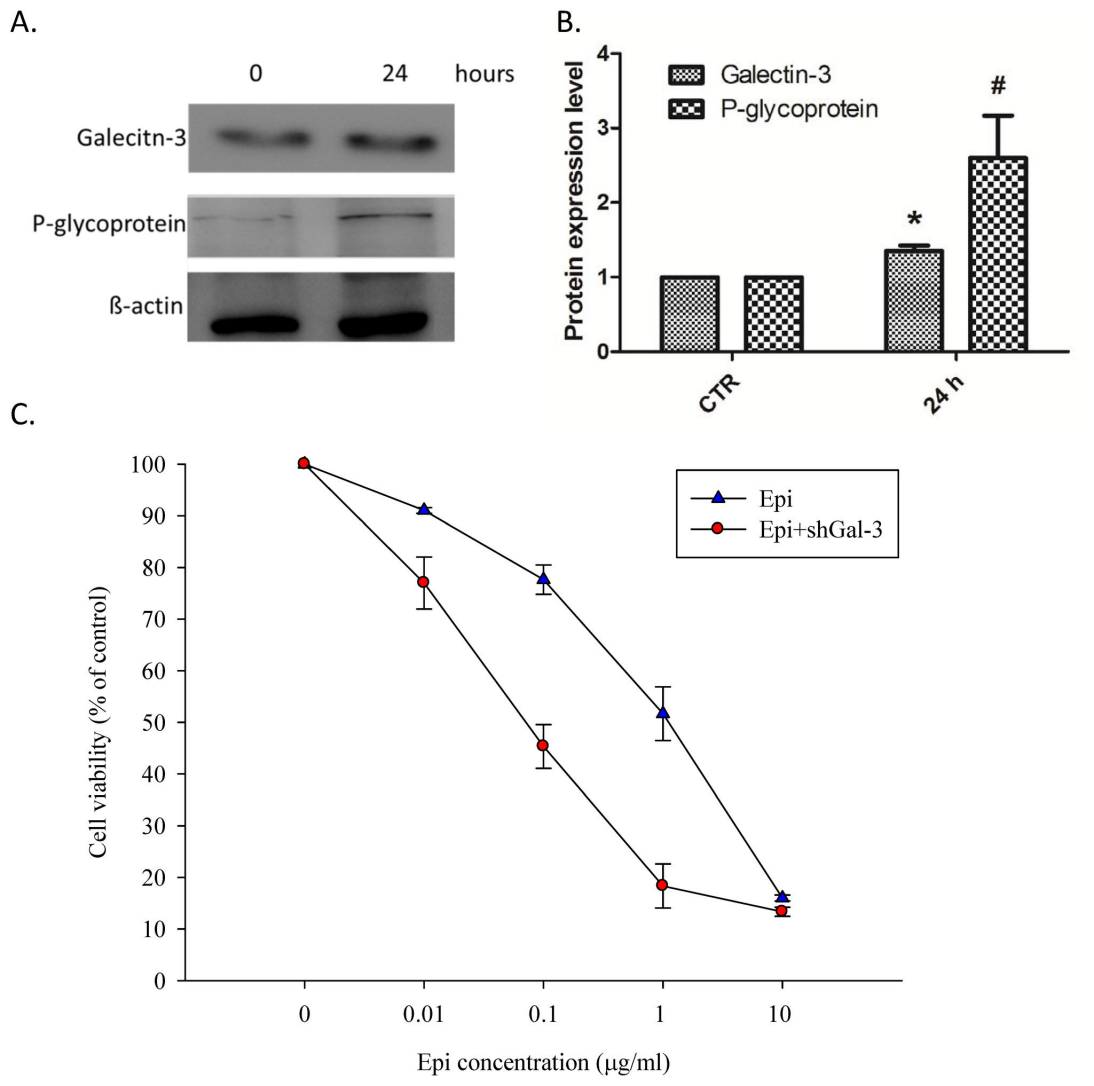
Galectin-3 expression and susceptibility of Caco-2 cells after epirubicin treatment. (**A**) Western blotting and (**B**) quantification results of Western blotting for galectin-3, P-glycoprotein (P-gp) and β-actin in Caco-2 cells after 1 μg/ml epirubicin (Epi) treatment for 24 h. Epirubicin treatment increased the expression of galectin-3 and P-gp. (**C**) Dose dependent effect of Epi on Caco-2 cells after a 48 h Epi treatment with or without shGal-3. The data are representative of at least three independent experiments. ^*, #^, *P* < 0.05 compared with the control (CTR).

First, Caco-2 cells were treated with epirubicin for 24 h, and the expression of galectin-3 and P-gp was analyzed by western blotting. As shown in Figures 1A and 1B, epirubicin significantly increased the expression of galectin-3 and P-gp. We then established a galectin-3 stable knockdown cell line by transfecting Caco-2 cells with shGal-3 for further studies. After 48 h treatment of the control and shGal-3 cells with increasing concentrations of epirubicin (0, 0.01, 0.1, 1, and 10 μg/ml), the relative cell viability (%) of the Caco-2 cells was evaluated with an MTT assay (Figure 1C). The epirubicin potency for cell-growth inhibition was expressed as the IC_50_ value. The mean IC_50_ value for epirubicin treatment was 0.91 ± 0.03 μg/ml. In addition, the IC_50_ value for the combined epirubicin and shGal-3 treatment was 0.07 ± 0.01 μg/ml, which is remarkably lower than that for epirubicin treatment alone. This combination enhanced epirubicin cytotoxicity and allowed for a reduced epirubicin dose.

### Combined epirubicin and shGal-3 treatment induced apoptosis-mediated morphological changes in Caco-2 cells and increased the sub-G_1_ accumulation of Caco-2 cell cycle

**Figure 2:**
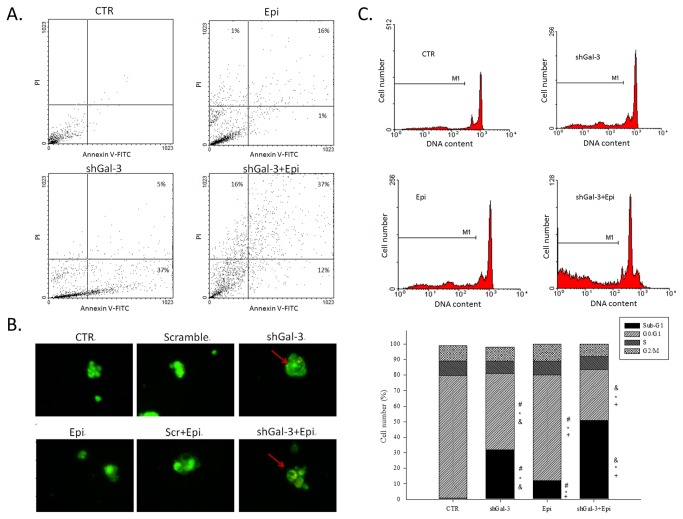
Epirubicin treatment induces apoptosis in galectin-3 knockdown calls. Cells were treated with medium, Epi, and/or shGal-3 for 48 h. (**A**) Cell apoptosis was then analyzed with a flow cytometer using annexin V/PI staining methods. The data are representative of three independent experiments and the numbers in the respective quadrants indicate the percentage of cells present in this area. (**B**) The cells were visualized with an inverted microscope equipped with a fluorescence image acridine orange. (**C**) Cell cycle was analyzed using flow cytometry. M1 represents the ratio of the number of cells in sub-G1 population to the number of total cells. The means ± SD from four independent measurements are shown. ^*^, *P* < 0.05 compared with the control (CTR); ^+^, *P* < 0.05 compared with shGal-3; ^&^, *P* < 0.05 compared with Epi.

Flow cytometric analysis using annexin V/PI staining showed that cells treated with epirubicin or shGal-3 alone exhibited a significant increase in apoptosis compared with the control (Figure 2A). The epirubicin and shGal-3 combination treatment remarkably promoted apoptosis with an increased amount of positive staining for membrane asymmetry and PI uptake (Figure 2A).

Caco-2 cells were treated with shGal-3 and/or 1 μg/ml epirubicin for 48 h and observed by fluorescence microscopy. The viable control cells and cells treated with scrambled shRNA had consistently bright green nuclei (Figure 2B). Caco-2 cells exposed to shGal-3 and/or epirubicin displayed plasma membrane blebbing, cell shrinkage, and concentrated green areas of condensed or fragmented chromatin in the nucleus (Figure 2B). The appearance of apoptotic bodies after shGal-3 and/or epirubicin treatment along with the apoptosis detection assay further verified that the cytotoxic effects of epirubicin alone or the combined shGal-3 treatment was mediated by apoptosis induction.

Caco-2 cells treated with shGal-3 and/or epirubicin exhibited a typical cell cycle phase distribution when examined with a flow cytometer. The proportion of cells exhibiting sub-G_1_ fluorescence, which corresponds to the apoptotic cell sub-population, significantly increased after treatment with shGal-3 and/or epirubicin for 48 h (Figure 2C). The percentage of the sub-G1 phase cells after combined treatment (51.3 ± 4.2%; *P* < 0.05) was significantly higher than that for cells treated with epirubicin (12.1 ± 1.4%) or shGal-3 (32.2 ± 1.1%). Thus, the silencing of galectin-3 by RNAi improved the ability of epirubicin-induced apoptosis in Caco-2 cells.

### shGal-3 significantly increased the intracellular epirubicin retention in Caco-2 cells

**Figure 3:**
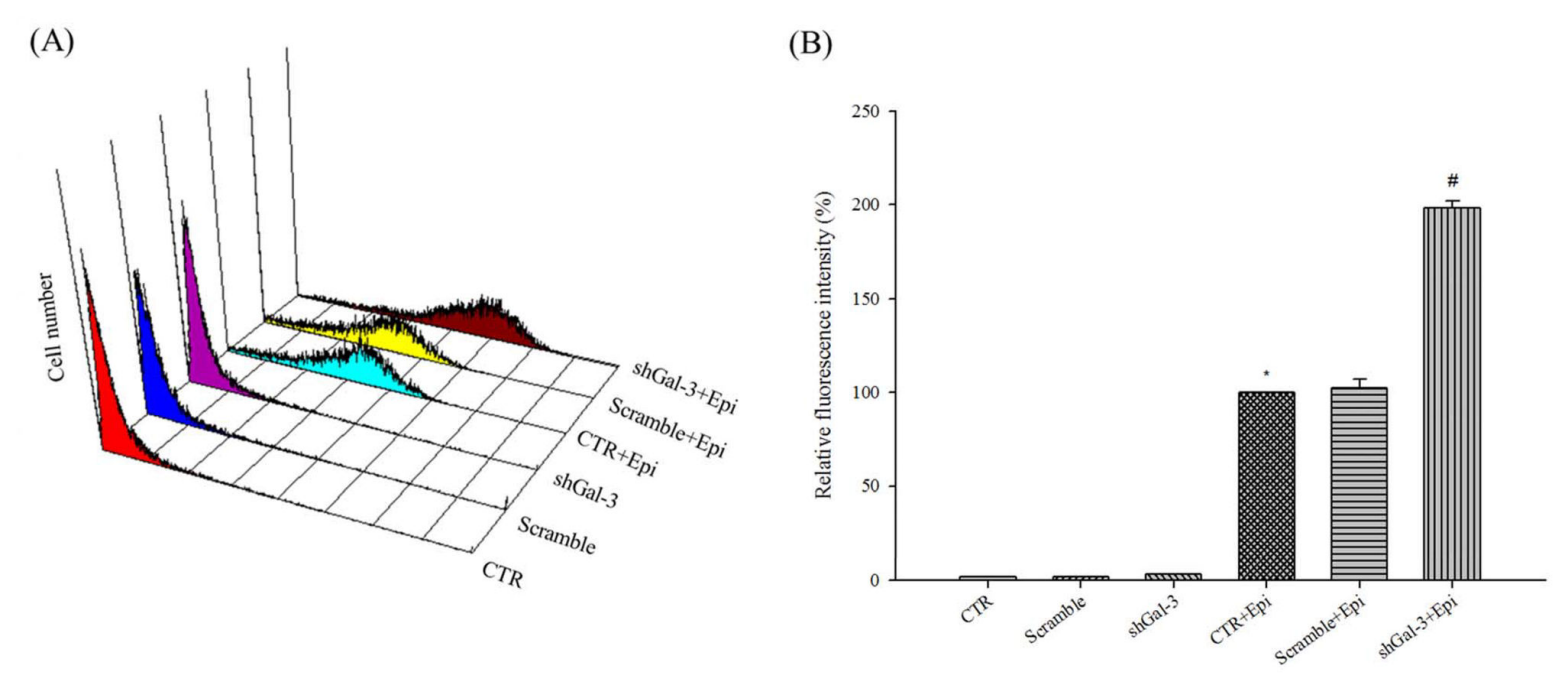
Knockdown of galectin-3 increases the intracellular epirubicin retention. Cells were pretreated with scrambled shRNA, shGal-3, and/or Epi for 3 h. (**A**) Three-dimensional view of cell number versus fluorescence intensity in Caco-2 cells. (**B**) The mean fluorescence intensity of Epi was normalized to 100%. The mean fluorescence intensity of Epi plus shGal-3 was normalized relative to Epi. *, *P* < 0.05 compared with control and #, *P* < 0.05 compared with Epi. The data are presented as the means ± SD from three independent experiments.

As pump-resistance efflux transporters, P-gp, MRP1, and MRP2 actively transport MDR substrates such as epirubicin out of cells and decrease the intracellular accumulation of epirubicin in cancer cells [3,22,24]. These phenomena mediate the development of pump-related MDR [25,26]. We found that the inhibition of pump resistance by shGal-3 treatment resulted in high epirubicin accumulation in Caco-2 cells as determined by flow cytometry (Figure 3A; P < 0.05). However, scrambled shRNA had no obvious effect on epirubicin retention in Caco-2 cells (Figure 3B).

### Combined shGal-3 and epirubicin treatment significantly inhibited the mRNA expression level of galectin-3 and β-catenin but increased the expression of GSK-3β

**Figure 4:**
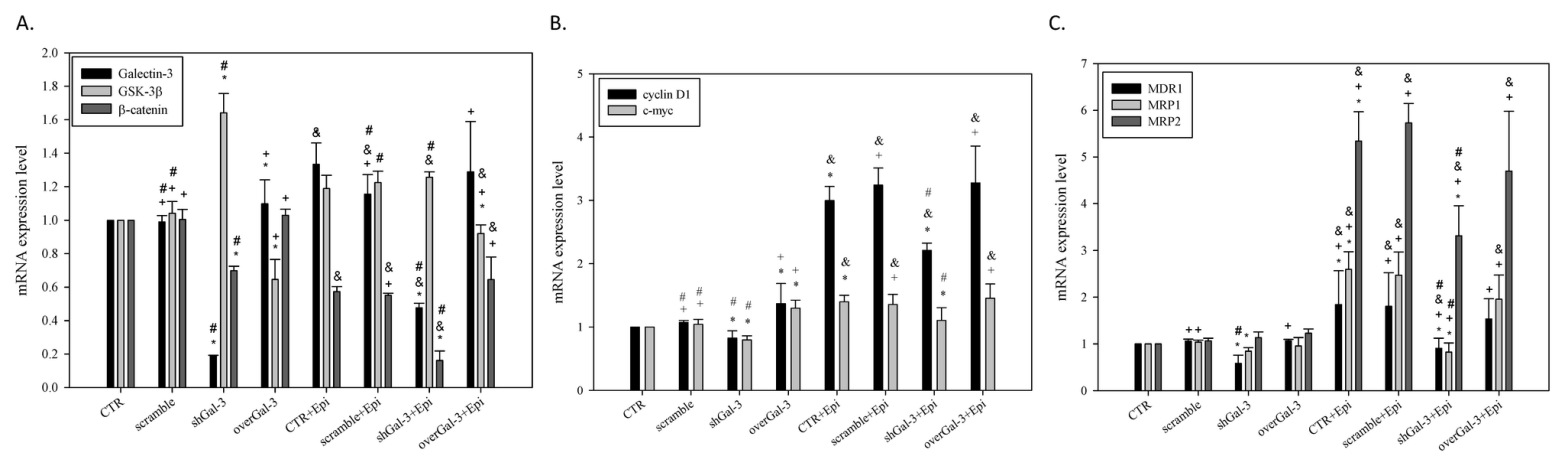
Knockdown of galectin-3 changes the mRNA expression patterns in Caco-2 cells after epirubicin treatment. (**A**) The effect of treatment with CTR, scrambled shRNA, shGal-3, transfection with a galectin-3 overexpression vector (overGal-3), and/or Epi for 48 h on the mRNA expression level of galectin-3, GSK-3β, and β-catenin as determined by real-time PCR. *, *P* < 0.05 compared with CTR; ^+^, *P* < 0.05 compared with shGal-3; ^&^, *P* < 0.05 compared with Epi; #, P<0.05 compared with overGal-3. (**B**) The effect of treatment with CTR, scrambled shRNA, shGal-3, overGal-3, and/or Epi for 48 h on the mRNA expression level of cyclin D1 and c-myc as determined by real-time PCR. *, *P* < 0.05 compared with CTR; ^+^, *P* < 0.05 compared with shGal-3; ^&^, *P* < 0.05 compared with Epi; and #, P<0.05 compared with overGal-3. (**C**) The effect of treatment with CTR, scrambled shRNA, shGal-3, overGal-3, and/or Epi for 48 h on the mRNA expression levels of the MDR pump-related genes encoding MDR1, MRP1, and MRP2 as determined by real-time PCR. *, *P* < 0.05 compared with CTR; ^+^, *P* < 0.05 compared with shGal-3; ^&^, *P* < 0.05 compared with Epi; #, P<0.05 compared with overGal-3.

Cells treated with shGal-3 inhibited the mRNA expression level of galectin-3 and β-catenin but increased the expression of GSK-3β (Figure 4A). The same trend could also be observed for the combined treatment (i.e., shGal-3 and epirubicin). In contrast, the transfection of a galectin-3 overexpression vector into Caco-2 cells decreased the GSK-3β mRNA level but slightly increased the mRNA level of galectin-3 and β-catenin. We also found that epirubicin treatment increased the mRNA expression of galectin-3 and GSK-3β but decreased the expression of β-catenin. These results indicated that the expression of galectin-3 and β-catenin mRNA in Caco-2 cells was upregulated and downregulated, respectively, with epirubicin treatment. In addition, the knockdown of galectin-3 upregulated the GSK-3β mRNA level, which was accompanied by the downregulation of β-catenin.

### Combined shGal-3 and epirubicin treatment inhibited the mRNA expression level of the cyclin D1 and c-myc induced by the epirubicin treatment

We further investigated the expression of the Wnt signaling-related genes cyclin D1 and c-myc upon galectin-3 knockdown and/or epirubicin treatment. The knockdown of galectin-3 slightly decreased the mRNA expression level of cyclin D1 and c-myc (*P* < 0.05; Figure 4B), whereas epirubicin significantly upregulated their expression (*P* < 0.05; Figure 4B). However, the combined treatment effectively reduced the mRNA expression of cyclin D1 and c-myc, which had been remarkably induced by epirubicin treatment. Moreover, the mRNA expression level of cyclin D1 induced by the combined treatment was higher than that of the control (*P* < 0.05; Figure 4B), but it was significantly lower than that for epirubicin treatment alone (*P* < 0.05). These results imply that cyclin D1-mediated epirubicin resistance is partially reversed by combined treatment (Figure 4B). The mRNA expression level of c-myc almost returned to the level of the control after combined treatment. Finally, these phenomena could be rescued after transfection with a galectin-3 overexpression vector.

### Combined shGal-3 and epirubicin treatment significantly inhibited the mRNA expression level of MDR1, MRP1, and MRP2

Next, we tried to understand whether the mRNA expression of the epirubicin-induced pump resistance proteins MDR1, MRP1, and MRP2 were regulated by galectin-3. We found that the corresponding mRNA expression level of MDR1, MRP1, and MRP2 was significantly increased by epirubicin treatment (*P* < 0.05), whereas that of MDR1 and MRP1 (*P* < 0.05) was moderately reduced after galectin-3 knockdown (Figure 4C). The reduction in the MDR1, MRP1, and MRP2 mRNA expression level was also observed for the combined treatment (Figure 4C, P < 0.05) and could be reversed after galectin-3 overexpression. These findings indicate that epirubicin-induced mRNA expression of MDR1, MRP1, and MRP2 is regulated by galectin-3 (Figure 4C).

### Combined shGal-3 and epirubicin treatment changed the mRNA expression level of Bax, Bcl-2, and caspases

**Figure 5:**
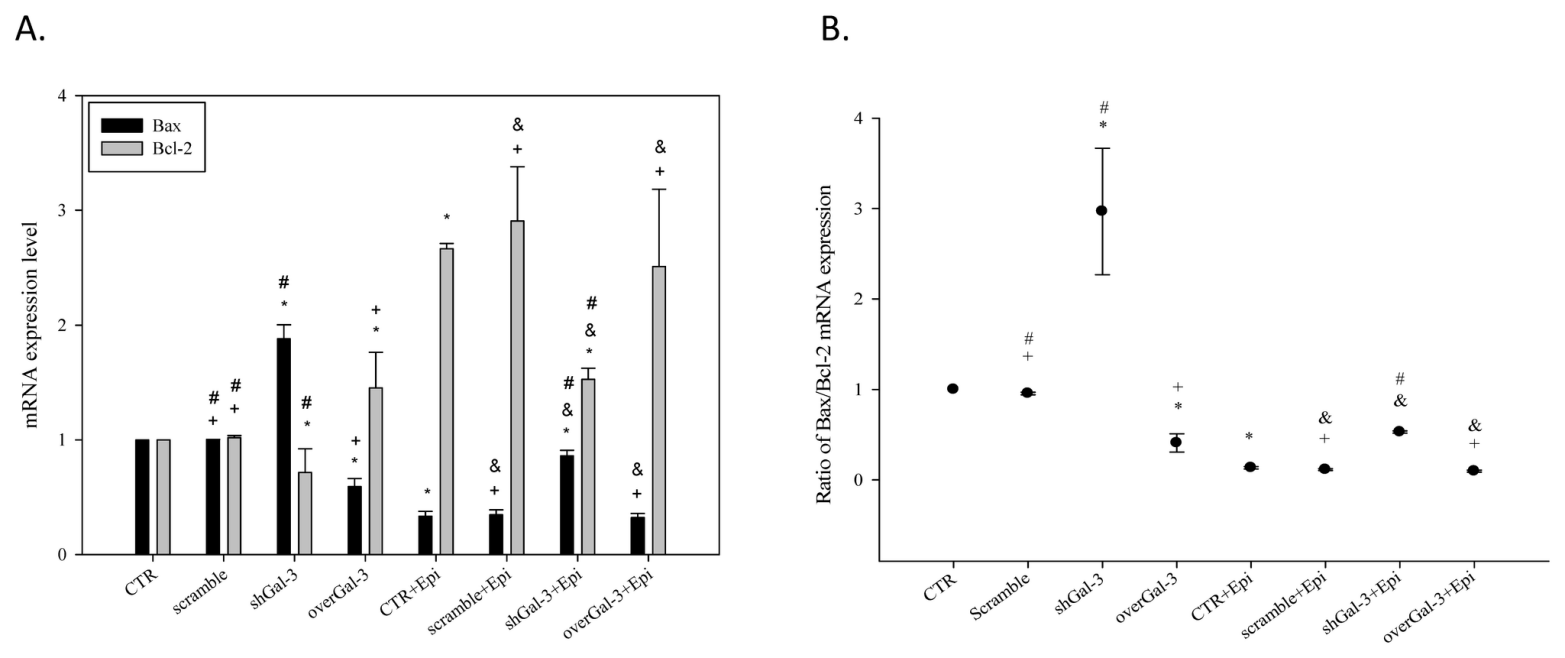
Knockdown of galectin-3 changes the mRNA expression levels of Bax, Bcl-2, and caspases after epirubicin treatment. (**A**) The effect of treatment with CTR, scrambled shRNA, shGal-3, overGal-3, and/or Epi (1 µg/ml) for 48 h on the mRNA expression level of the apoptosis-related genes encoding Bcl-2 and Bax as determined by real-time PCR. (**B**) The effect of different treatments on the ratio between the Bax:Bcl-2 mRNA expression. *, *P* < 0.05 compared with CTR; ^+^, *P* < 0.05 compared with shGal-3; ^&^, *P* < 0.05 compared with Epi; #, P<0.05 compared with overGal-3.

**Figure 6:**
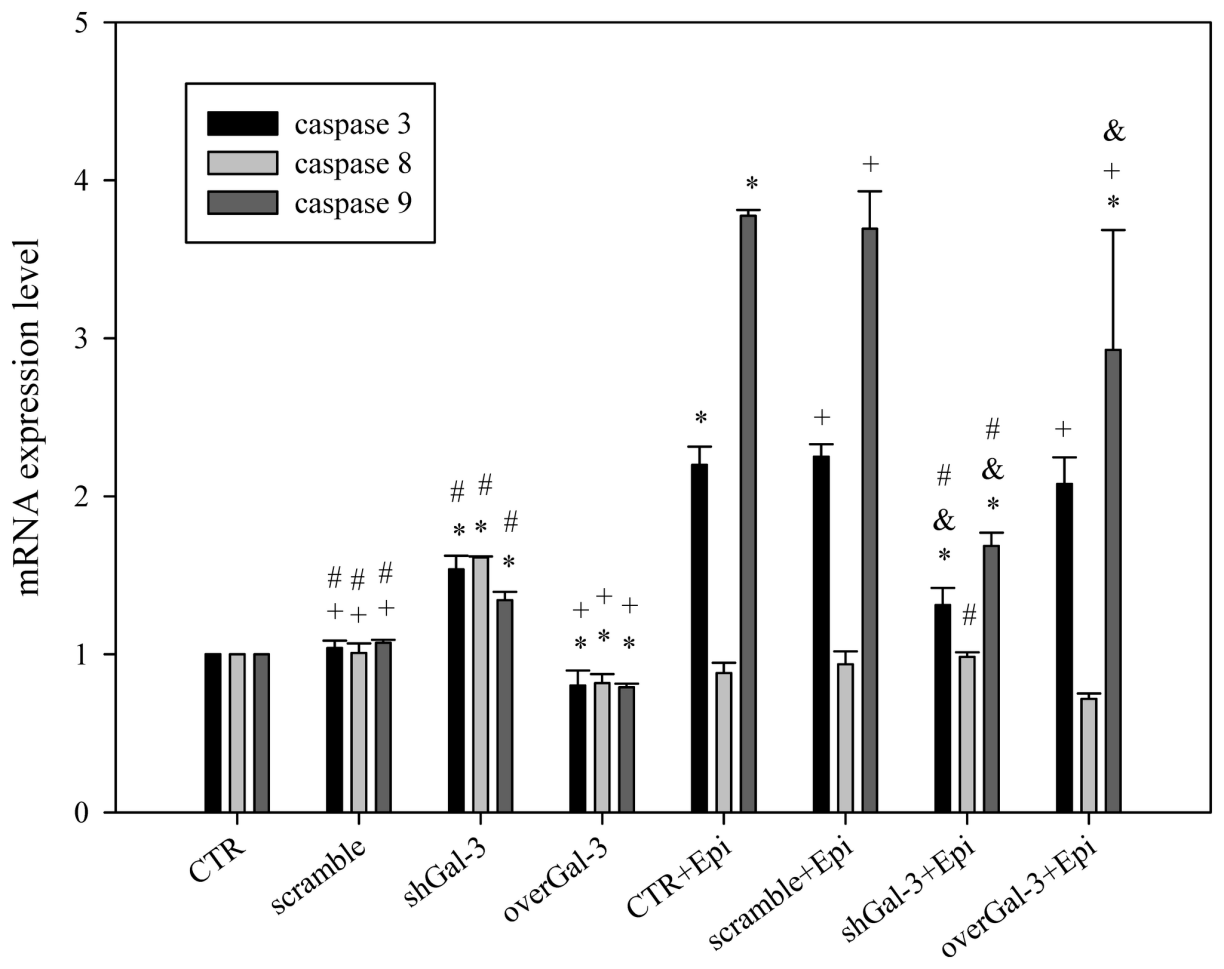
Knockdown of galectin-3 changes the mRNA expression levels of caspase-3, 8 and 9 after epirubicin treatment. The effect of treatment with CTR, scrambled shRNA, shGal-3, overGal-3, and/or Epi for 48 h on the mRNA expression level of the apoptosis-related genes encoding caspase-3, caspase-8, and caspase-9 as determined by real-time PCR. *, *P* < 0.05 compared with CTR; ^+^, *P* < 0.05 compared with shGal-3; ^&^, *P* < 0.05 compared with Epi; #, P<0.05 compared with overGal-3.

In addition, we examined the expression of Bax, Bcl-2 and caspases upon shGal-3 and/or epirubicin treatment. The knockdown of galectin-3 increased Bax and reduced Bcl-2 mRNA expression, which leads to apoptosis (Figure 5A; P < 0.05). In contrast, the overexpression of galectin-3 reversed these phenomena. Treatment with epirubicin alone significantly modulated the corresponding mRNA level of Bax, caspase-3, and caspase-9 (Figure 5A and 6; *P* < 0.05). In addition, the knockdown of galectin-3 remarkably increased the Bax-to-Bcl-2 ratio (Figure 5B; P < 0.05). However, due to the development of resistance, epirubicin reduced the expression of Bax and increased the level of Bcl-2. Furthermore, the knockdown of galectin-3 partially reversed epirubicin-mediated resistance.

### Confirm the protein expression level of the Wnt/β-catenin signaling pathway and apoptosis-associated pathway components after epirubicin treatment in wild type or galectin-3 knockdown Caco-2 cells

**Figure 7:**
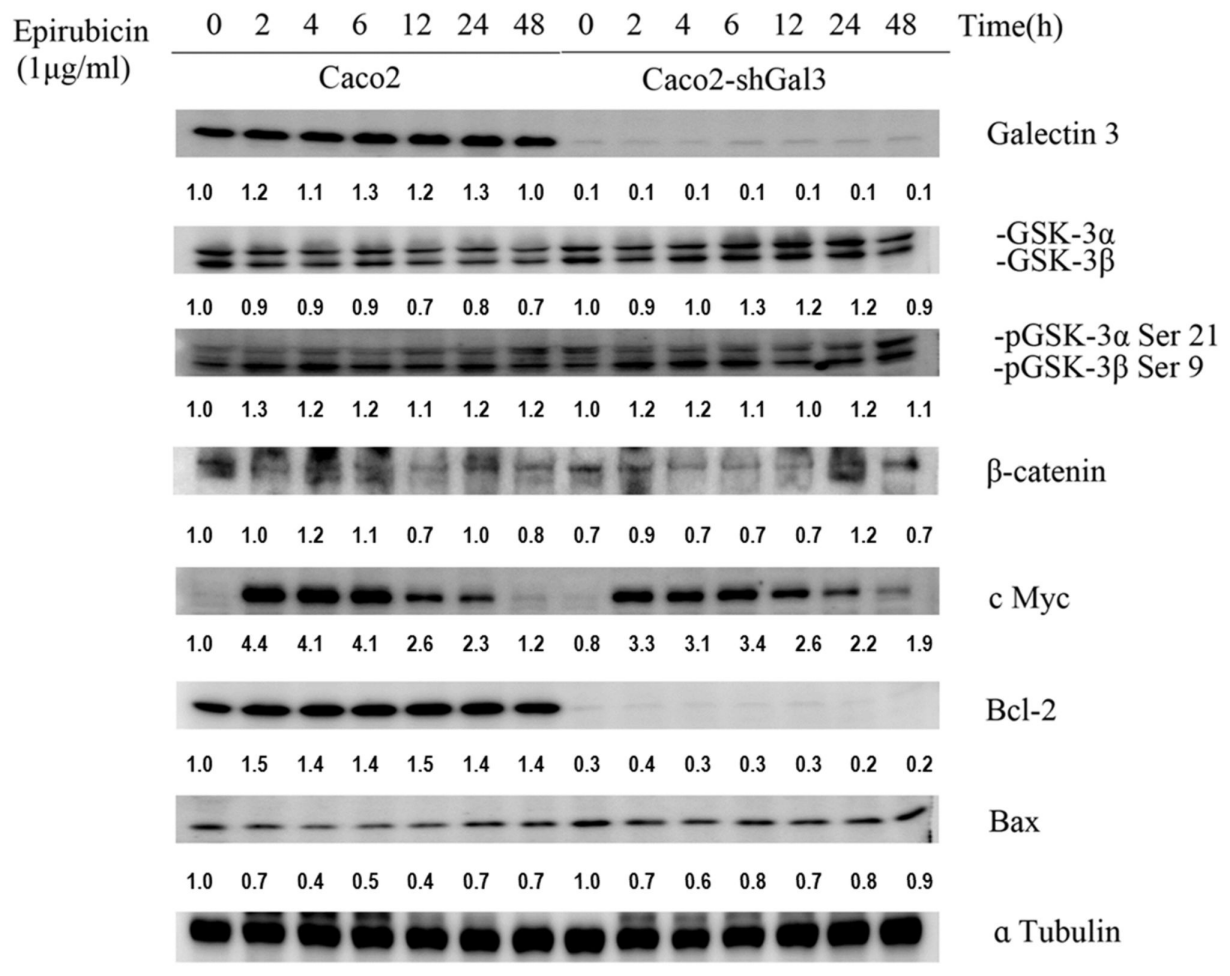
Knockdown of galectin-3 changes the protein expression of the Wnt/β-catenin signaling and apoptosis-associated pathways after epirubicin treatment. GSK-3β and Bax were upregulated after the treatment of galectin-3 knockdown cells. The expression of the anti-apoptotic protein Bcl-2 was significantly reduced in galectin-3 knockdown cells. The simultaneous downregulation of pGSK-3β, β-catenin, c Myc, and the pro-apoptotic protein Bcl-2 was also found.

 To verify the results observed in real-time PCR, we further examined the expression level of Wnt signaling pathway and apoptosis-associated proteins by western blotting (Figure 7). In accordance with previous findings, GSK-3β and Bax were upregulated after the treatment of galectin-3 knockdown cells with epirubicin for 48 h. The simultaneous downregulation of pGSK-3β, β-catenin, c Myc, and the pro-apoptotic protein Bcl-2 was also found. In addition, the expression of the anti-apoptotic protein Bcl-2 was significantly reduced in galectin-3 knockdown cells. It should be noted that the upregulation of GSK-3β and Bax and the down regulation of pGSK-3β, β-catenin, c Myc, and Bcl-2 could also be found at 2, 4, 6, 12, and 24 h after epirubicin treatment (Figure 7).

### The knockdown of galectin-3 in Caco-2 cells inhibited the P-g and cyclin-D1 protein expression after epirubicin treatment

**Figure 8:**
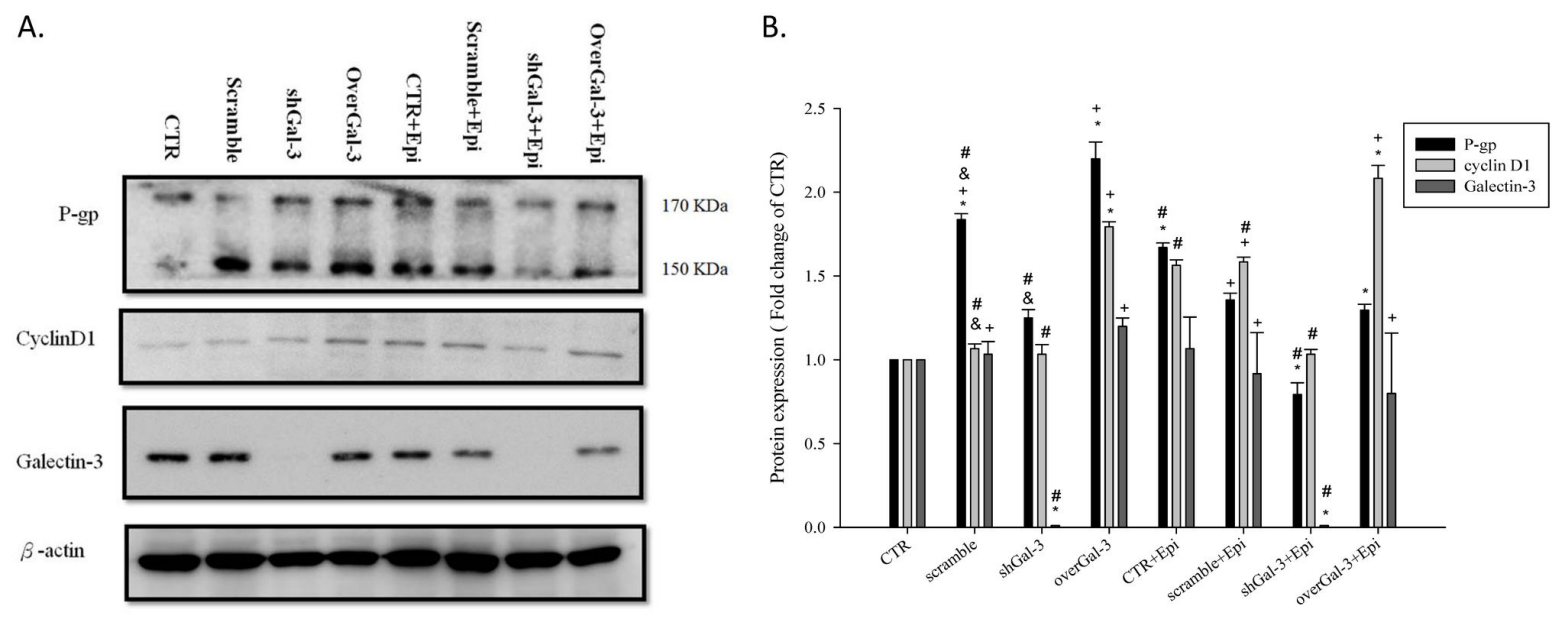
Knockdown of galectin-3 changes the protein expression of galectin-3, cyclin D1, and P-gp after epirubicin treatment. The effect of treatment with CTR, scrambled shRNA, shGal-3, overGal-3, and/or Epi for 48 h on the protein expression of galectin-3, cyclin D1, and P-gp as determined by western blot assay. *, *P* < 0.05 compared with CTR; ^+^, *P* < 0.05 compared with shGal-3; ^&^, *P* < 0.05 compared with Epi; #, P<0.05 compared with overGal-3.

 Because activation of the β-catenin signaling pathway by GSK-3β inhibition can increase the expression of P-gp, we knocked down or overexpressed the upstream regulator galectin-3 to decrease the GSK-3β activity by transfecting Caco-2 cells with galectin-3 siRNA or a galectin-3 expression vector. We found that the P-gp expression was concomitantly increased with the elevation of cyclin D1 in galectin-3 overexpressing cells. The same results could also be observed in epirubicin-treated cells. In addition, The knockdown of galectin-3 could reduce the expression of cyclin D1 and the downstream product P-gp (Figure 8).

### Combined shGal-3 and epirubicin treatment increased the caspase-3 and caspase-9 activity

**Figure 9:**
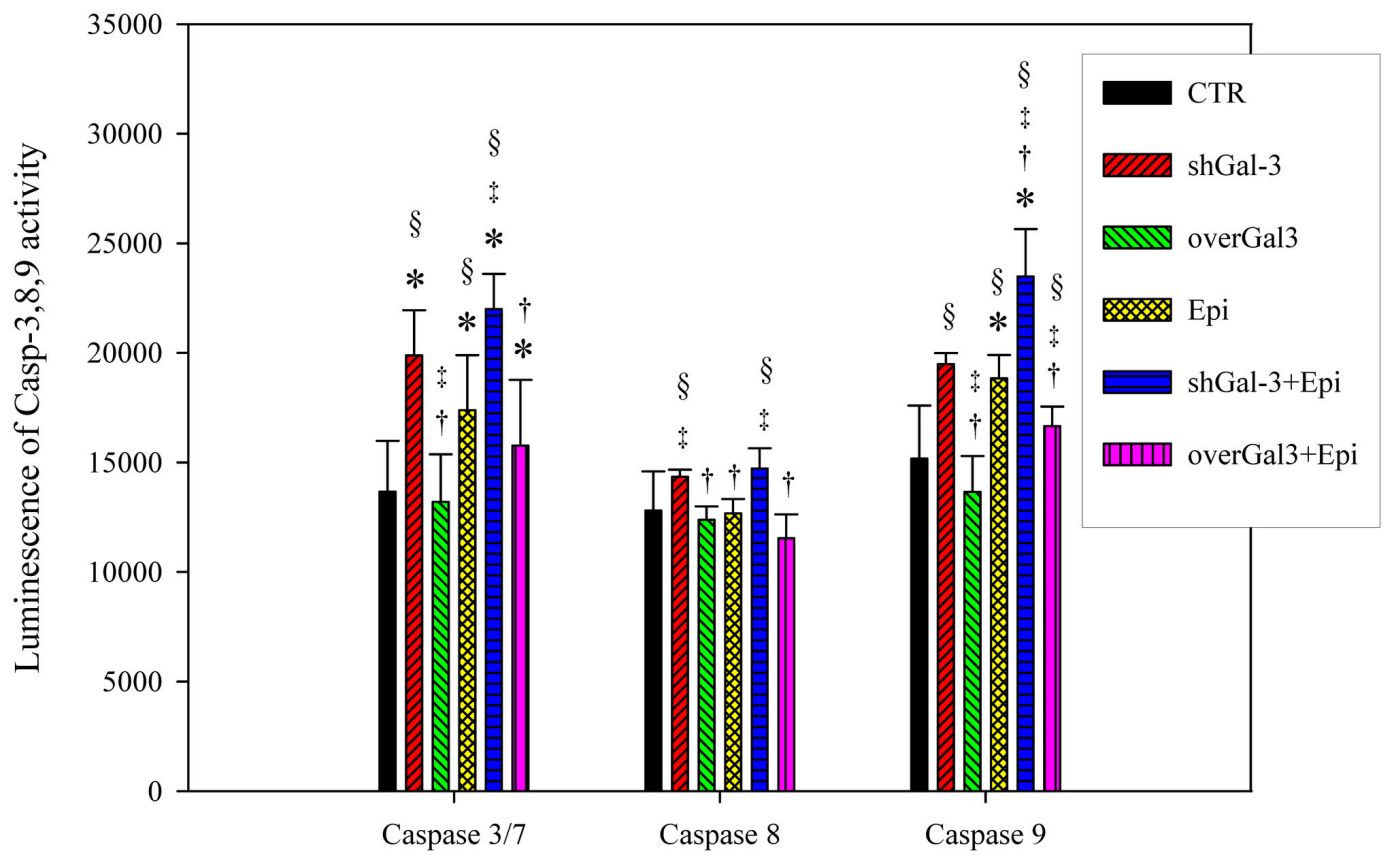
Knockdown of galectin-3 changes the activity of caspase 3, 8 and 9 after epirubicin treatment. The effect of treatment with CTR, scrambled shRNA, shGal-3, overGal-3, and/or Epi for 48 h on caspase-3, caspase-8, and caspase-9 activity levels in Caco-2 cells. Data are presented as the means ± SD from three independent experiments. ^*^, *P* < 0.05 compared with CTR; ^†^, *P* < 0.05 compared with shGal-3; ^‡^, *P* < 0.05 compared with Epi; ^§^, *P* < 0.05 compared with overGal-3.

Finally, we evaluated the activity of the caspase-3, caspase-8, and caspase-9 induced in galectin-3 knockdown cells with or without epirubicin treatment to determine the apoptotic pathway involved. The activity of caspase-3 and caspase-9 was enhanced by shGal-3 or epirubicin alone treatment (*P* < 0.05) and further increased by the combined treatment (Figure 9; *P* < 0.05). However, the activity of caspase-8 was not influenced by galectin-3 knockdown, epirubicin, or combined treatment.

## Discussion

P-gp and MRPs are involved in increasing tumor cell survival and delaying the apoptosis cascade [27,28]. Overexpressed P-gp and MRPs such as MRP1 and MRP2, members of ABC transporters, are the main causes of pump-related MDR. TCF4/β-catenin responsive elements are found in the promoter of the human *MDR1* gene, corresponding to a positive correlation between the expression of β-catenin, c-myc, and cyclin D1 and the upregulation of P-gp [29-31]. c-Myc can activate *MDR1* transcription by binding to the E-box motif located in the *MDR1* gene promoter, thus positively controlling MDR expression [32]. Knockdown of c-myc significantly downregulates c-myc and P-gp expression and results in an increase in doxorubicin uptake [33].The expression of *MDR1* can also be upregulated by glucosylceramide synthase (GCS) through the β-catenin signaling pathway [34], and a reduction in the GCS activity by inhibitors can induce GSK-3-regulated apoptosis via the accumulation of endogenous ceramide [35]. Phosphorylation of GSK-3β at serine 9 inactivates GSK-3β activity and causes the stabilization and activation of β-catenin signaling, which subsequently enhances P-gp expression [31,36]. Furthermore, the stable transfection of a cyclin D1 antisense construct into human pancreatic cancer cells leads to decreased levels of the mRNA of chemoresistance genes such as *MDR1* and *MRP* [37].

The upregulation of the antiapoptotic survival defensive system is one of the primary non-pump related MDR mechanisms. Although the anti-apoptotic role and mechanism of galectin-3 in cancer drug resistance have been studied [38], the regulatory pathway and mechanism(s) of chemoresistance-associated proteins modulated by galectin-3 in MDR cancer cells have not been fully elucidated. Previous reports have shown that galectin-3 mediates nuclear β-catenin accumulation and subsequently increases TCF4 transcriptional activity followed by the upregulation of its target genes, such as cyclin D1 and c-myc, in colon cancer cells [9,19]. Yamamoto-Sugitani et al. also showed that high level of galectin-3 expression in chronic myelogenous leukemia prompted drug resistance through activation of Akt and Erk [39]. Knockdown of galectin-3 inhibits phosphorylation of GSK-3β and increases GSK-3β expression, which leads to β-catenin phosphorylation and results in the degradation of β-catenin by the proteasome [19,20,40]. In addition, GSK-3β is also involved in cyclin D1 mRNA transcription and ubiquitin-dependent proteolysis [41].

In this study, we found that Caco-2 cells reduced epirubicin-induced apoptosis not only through an increase in galectin-3 expression but also through the activation of P-gp (Figure 1). The reduced expression of galectin-3 significantly enhanced the cytotoxicity of the epirubicin treatment (Figure 1) and increased apoptosis (Figure 2). Western blotting and real-time PCR results showed that galectin-3 silencing resulted in decreased levels of phospho-GSK-3β at serine 9 and increased GSK-3β and GSK-3α expression. The increased expression of GSK-3β led to the downregulation of β-catenin and, subsequently, cyclin D1 and c-myc. These findings indicated that the knockdown of galectin-3 inhibits the expression of Wnt downstream effectors including β-catenin, cyclin D1 and c-myc at the mRNA and protein levels. We also found that the combined treatment of epirubicin and shGal-3 efficiently reversed the MDR transporter-mediated resistance induced by epirubicin administration as demonstrated by epirubicin accumulation, real-time PCR, western blotting, and MTT assays. These data suggest that shGal-3 downregulates the expression of cyclin D1 and c-myc, which play important roles as oncogenes and MDR/MRPs transcriptional upregulators. In accordance with these observations, the silencing of galectin-3 effectively inhibits the expression of MDR1, MRP1, and MRP2, thus circumventing pump-resistance and enhancing the sensitivity of Caco-2 cells to chemotherapy. This is the first demonstration showing that the silencing of galectin-3 may inhibit MDR through the simultaneous suppression of ABC transporters, such as P-gp, MRP1, and MRP2 via the β-catenin/GSK-3β pathway. 

In addition to understanding the role of galectin-3 in cancer drug resistance, we further examined the key role of shGal-3 in apoptosis induction. Cheong et al. have reported that silencing of galectin-3 augmented apoptosis induction with chemotherapy by decreasing the expression of cell survival molecules such as survivin and cyclin D1 in gastric cancer cells [42]. Suppression of cyclin D1 has also been demonstrated to inhibit tumor cell growth in human hepatocellular carcinoma and colonic adenocarcinoma [43]. Our findings demonstrated that shGal-3 and/or epirubicin treatment triggered the apoptosis of Caco-2 cells. The combined treatment of epirubicin and shGal-3 exhibited a better apoptosis-inducing effect than that of epirubicin and shGal-3 treatment alone. Galectin-3 silencing interrupted the β-catenin pathway and inhibited cyclin D1 and c-myc, which play critical roles in cell cycle regulation. Consistent with these models, our results support the idea that the suppression of cyclin D1 and c-myc inhibits the growth of Caco-2 cells and increases their chemosensitivity to epirubicin.

**Figure 10:**
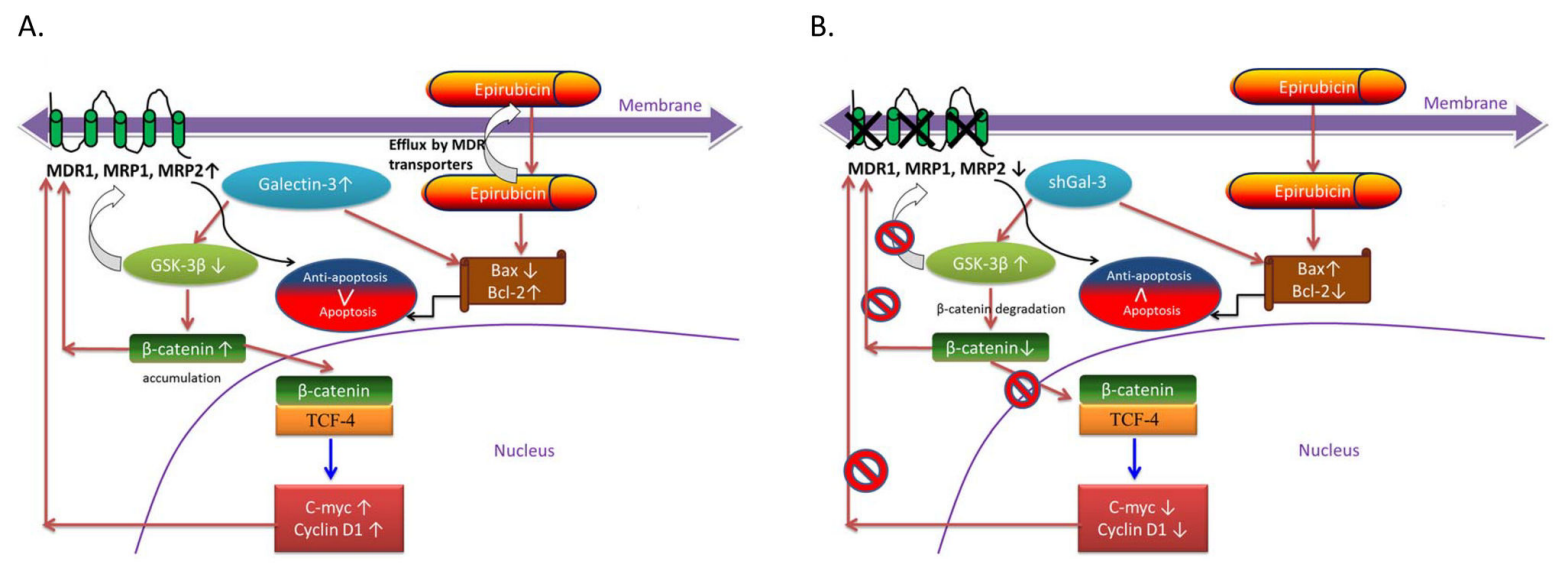
Proposed schematic diagram for the possible mechanisms of reversing pump and non-pump MDR in Caco-2 cells.

The caspase family of cysteine proteases subsequently mediated an apoptosis cascade corresponding to changes in the cell cycle and morphological observations as determined with a flow cytometer and fluorescence microscope. Caspase-9 is an initiator caspase for the mitochondrial pathway whereas caspase-8 is an initiator caspase for the death receptor pathway, and both of these pathways share downstream effector caspases such as caspase-3 and caspase -7 [3,23]. According to our findings, shGal-3 and/or epirubicin treatment triggered apoptosis through the intrinsic mitochondrial pathway (a negligible change in caspase-8), which is in agreement with previous studies [3,22,23]. The proposed scheme of molecular mechanisms involved in galectin-3 silencing is shown in Figure 10.

In conclusion, we show for the first time that galectin-3 silencing sensitizes MDR cells to epirubicin by inhibiting P-gp and MRPs and the activation of the mitochondrial apoptosis pathway through modulation of the β-catenin/GSK-3β pathway in human colon cancer cells. The complicated regulation of MDR highlights the need for a multifunctional approach for the combination of epirubicin and shRNAs directed against galectin-3 for which upstream regulatory signaling efflux pump proteins and complex apoptosis pathways are simultaneously targeted to make significant improvement in the clinical efficacy of anticancer therapy.
