# Correction: Galectin-3 Silencing Inhibits Epirubicin-Induced ATP Binding Cassette Transporters and Activates the Mitochondrial Apoptosis Pathway via β-Catenin/GSK-3β Modulation in Colorectal Carcinoma

**DOI:** 10.1371/journal.pone.0107784

**Published:** 2014-09-08

**Authors:** The PLOS ONE Staff

There are errors in Table 1. The primer sequences listed are in correct. Please see the corrected Table 1 here.

**Table 1:** The sequences of shRNA and primer sequences used for knock-down and real-time PCR.

Gene name	Sequences
shGal-3 target sequence	5’-GCTCACTTGTTGCAGTACAAT-3’
shLacZ target sequence	5’-TGTTCGCATTATCCGAACCAT-3’
Gal-3 forward primer	5’-TAATAACTGGGGAAGGGAAG-3’
Gal-3 reverse primer	5’-AGCACTGGTGAGGTCTATGT-3’
GSK-3β forward primer	5’-ACTTTGTGACTCAGGAGAACTGG-3’
GSK-3β reverse primer	5’-TCGCCACTCGAGTAGAAGAAATA-3’
β-catenin forward primer	5’-TCAGATCTTAGCTTACGGCAA-3’
β-catenin reverse primer	5’-TTGTTGCTAGAGCAGACAGAC -3’
Cyclin D1 forward primer	5’-CACACGGACTACAGGGGAGT-3’
Cyclin D1 reverse primer	5’-CACAGGAGCTGGTGTTCCAT-3’
C-myc forward primer	5’-TCAAGAGGCGAACACACAAC-3’
C-myc reverse primer	5’-GGCCTTTTCATTGTTTTCCA-3’
Bcl-2 forward primer	5’-CTTGACAGAGGATCATGCTGTAC-3’
Bcl-2 reverse primer	5’-GGATGCTTTATTTCATGAGGC-3’
Bax forward primer	5’-GGGCCCACCAGCTCTGA-3’
Bax reverse primer	5’-CCTGCTCGATCCTGGATGA-3’
Caspase 3 forward primer	5’-CCTGGTTATTATTCTTGGCGAAA-3’
Caspase 3 reverse primer	5’-GCACAAAGCGACTGGATGAA-3’
Caspase 8 forward primer	5’-CAGGCAGGGCTCAAATTTCT-3’
Caspase 8 reverse primer	5’-TCTGCTCACTTCTTCTGAAATCTGA-3’
Caspase 9 forward primer	5’-TGCTGAGCAGCGAGCTGTT-3’
Caspase 9 reverse primer	5’-AGCCTGCCCGCTGGAT-3’
18s rRNA forward primer	5’-CAGAATCCACGCCAGTACAA-3’
18s rRNA reverse primer	5’-AATCTTCTTCAGTCGCTCCA-3’
